# Preliminary study on the effect of catabolite repression gene knockout on *p*-nitrophenol degradation in *Pseudomonas putida* DLL-E4

**DOI:** 10.1371/journal.pone.0278503

**Published:** 2022-12-02

**Authors:** Shuang Li, Yichao Tang, Lingran Tang, Xuanyu Yan, Jiali Xiao, Huijun Xiang, Qing Wu, Ruqi Yu, Yushi Jin, Jingyu Yu, Nuo Xu, Chu Wu, Shengqin Wang, Chuanhua Wang, Qiongzhen Chen

**Affiliations:** 1 College of Life and Environmental Science, Wenzhou University, Wenzhou, People’s Republic of China; 2 National and Local Joint Engineering Research Center for Ecological Treatment Technology of Urban Water Pollution, Wenzhou University, Wenzhou, People’s Republic of China; Ruhr-Universitat Bochum, GERMANY

## Abstract

*P*-nitrophenol (PNP) is a carcinogenic, teratogenic, and mutagenic compound that can cause serious harm to the environment. A strain of *Pseudomonas putida* DLL-E4, can efficiently degrade PNP in a complex process that is influenced by many factors. Previous studies showed that the expression level of *pnpA*, a key gene involved in PNP degradation, was upregulated significantly and the degradation of PNP was obviously accelerated in the presence of glucose. In addition, the expression of *crc*, *crcY*, and *crcZ*, key genes involved in catabolite repression, was downregulated, upregulated, and upregulated, respectively. To investigate the effect of the carbon catabolite repression (CCR) system on PNP degradation, the *crc*, *crcY*, and *crcZ* genes were successfully knocked out by conjugation experiments. Our results showed that the knockout of *crc* accelerated PNP degradation but slowed down the cell growth. However, the knockout of *crcY* or *crcZ* alone accelerated PNP degradation when PNP as the sole carbon source, but that knockout slowed down PNP degradation when glucose was added. The results indicate that the CCR system is involved in the regulation of PNP degradation, and further work is required to determine the details of the specific regulatory mechanism.

## Introduction

Nitrophenols are important aromatic compounds that are widely used in the production of pharmaceuticals, dyes, wood preservatives, rubber, explosives, and pesticides. *P*-nitrophenol (PNP) is one of the important nitrophenol pollutants, has carcinogenic, teratogenic, and mutagenic effects [1], and can enter the environment in multiple ways [2]. PNP has been placed on the list of priority pollutants to be controlled by environmental agencies in China and the United States [3]. The biodegradation of PNP mainly occurs through the 1,2,4-benzenetriol and hydroquinone pathways. There has been nearly 30 years of research on microbial biodegradation of PNP. Scientists have isolated a large number of microbial strains capable of degrading PNP, and analyzed their microbial metabolic pathways, degradation genes, proteins, etc. [4–13]. The *Pseudomonas putida* DLL-E4 strain used in this study can degrade PNP efficiently through the 1,2,4-benzenetriol and hydroquinone pathways [14].

We have conducted preliminary studies of the regulation of PNP degradation and found that *pnpA* and *pnpC1C2DECX1X2* are key genes of PNP degradation in *P*. *putida* DLL-E4, and these genes are positively regulated by PnpR, a transcriptional regulatory protein belonging to the LysR family [14]. PnpA, 4-nitrophenol monooxygenase, is encoded by *pnpA* in *P*. *putida* DLL-E4, and can catalyze the denitrification of PNP in the initial stage of PNP degradation. PnpA is a key enzyme required for PNP degradation, and its expression level directly affects the efficiency of PNP degradation. Our previous data showed that both the expression of *pnpA* and the degradation rate of PNP increased significantly in the presence of additional glucose. In this same condition, the expression of *crc*, a gene involved in the carbon catabolite repression (CCR) system, decreased significantly, and the expression of other CCR related genes, *crcY* and *crcZ*, increased significantly [14]. These findings and previous results in the literature [15–21] suggested that PNP degradation might be regulated by the CCR system.

Several groups have studied the biological function of Crc, CrcY, and CrcZ using proteomic, transcriptomic, lacZ fusion, or Real-Time PCR methods in *Pseudomonas* strains [18–21]. Moreno R. *et al*. [19] found that the inactivation of *crc* causes dramatic transcriptomic and proteomic changes, modifies the expression of at least 134 genes involved in the transport and assimilation of amino acids or sugars, leading to a decrease in growth rate [19]. Translation of the target gene can be inhibited by Crc when Crc combined with the target mRNA. However, noncoding RNAs *crcY* and *crcZ* can combine with Crc, leading to the elimination of target gene translation inhibition caused by Crc. This elimination then alleviate or even eliminate the phenomenon of catabolite repression [20, 21]. Double knockout of *crcY* and *crcZ* can result in the inability to relieve catabolite repression, thus affecting the ability of cells to utilize other carbon sources [21]. The CCR system regulates the metabolism of a variety of aromatic compounds, such as toluene [22], phenol [23], protocatechuic acid [24], and benzoic acid [25], however regulation of PNP degradation has not been reported. In this study, the *crc*, *crcY* and *crcZ* knock-out strains have been successfully screened by conjugation experiments. The effects of *crc*, *crcY*, and *crcZ* knock-out were investigated for PNP degradation in *P*. *putida* DLL-E4. The resulting strains are important genetic resources for future study, and can be used to investigate the specific regulatory mechanism of the CCR system on PNP degradation.

## Materials and methods

### Reagents, strains and plasmids

The reagents for polymerase chain reaction (PCR), restriction endonuclease digestion, and ligation used in this study were all purchased from TaKaRa. The chemical reagents were all purchased from Sinopharm group, and primer synthesis and sequencing were performed by Sangon Biotech (Shanghai) Co., Ltd. The primer sequences used in this study are listed in Table 1.

**Table 1:** Primers of this study.

Primers	Sequences (5’-3’)
**For the deletion of *crc* gene**	
*crc*-5F	TCGTTAACCTGTCCCGGCCTATTC
*crc*-5R	GTTCACACTGATGATCCGCATAAATGGC
*crc*-3F	CATGCGCCGCTGATTGTCGA
*crc*-3R	GTCGACTGCGACGTGATCCAG
*crc*-KnF	GCCATTTATGCGGATCATCAGTGTGAACGAGCTGCTTCGAAGTTCCTA
*crc*-KnR	TCGACAATCAGCGGCGCATGCATATGAATATCCTCCTTAGTTCCTATTC
*crc*-outF	GTGTGAATAGCTCCCACATCATCGAC
*crc*-outR	TGAGCAAGCTCGGCGATATCAC
*crc*-inF	CTTCGAGACAGCCGACCGCTAC
*crc*-inR	GATATCGAGCTTCTGCTGCGCCAC
Kn-seqF	GTATCCATCATGGCTGATGCAATGC
Kn-seqR	GCATTGCATCAGCCATGATGGATAC
**For the deletion of *crcY* gene**	
*crcY*-5F	GTGAAGGAAGTGGCGGTGTTC
*crcY*-5R	TGTTGTTGTACCAGAGATATAGCAGGG
*crcY*-3F2	GAGCAGGCAACAACGCTTTGA
*crcY*-3R2	CCATCACCCTCGATGCGCAG
*crcY*-KnF	CCCTGCTATATCTCTGGTACAACAACACATATGAATATCCTCCTTAGTTCCTATTC
*crcY*-KnR2	TCAAAGCGTTGTTGCCTGCTCGAGCTGCTTCGAAGTTCCTA
*crcY*-outF	GCCTATATCGAAGTGGGCAGTTTCG
*crcY*-outR	CGTCGGTGACGATTTCGATGGAC
*crcY*-inF	GAACAACACGGCAGAGGCGTAG
*crcY*-inR	CTGACCTGCTTTCATCATTGCTGATCC
Kn-seqF	GTATCCATCATGGCTGATGCAATGC
Kn-seqR	GCATTGCATCAGCCATGATGGATAC
**For the deletion of *crcZ* gene**	
*crcZ*-5F	CAGGCCATTCGTCACTACAGCT
*crcZ*-5R	TTCTTGTACCAGACATATAGCAGGTGC
*crcZ*-3F2	GACTTCTTGGGGAGCTTAGGCTC
*crcZ*-3R2	CGACCAGCATTTCGAAGACGATG
*crcZ*-GmF	GCACCTGCTATATGTCTGGTACAAGAAAGAAATGCCTCGACTTC
*crcZ*-GmR2	GAGCCTAAGCTCCCCAAGAAGTCTTGTGACAATTTACCGAACAAC
*crcZ*-outF	CGACGTCAACGAGATCGCCAG
*crcZ*-outR	GGCTGATCAGCGTGTGGGTG
*crcZ*-inF	CACAACCAGGCAGAGAACAAC
*crcZ*-inR	CACAAATCTCGAAGCTTGTGGAC
Gm-seqF	CAACATCAGCCGGACTCCGATTAC
Gm-seqR	GTAATCGGAGTCCGGCTGATGTT

Strains *P*. *putida* DLL-E4, *Escherichia coli* DH5α λ pir, *E*. *coli* DH10B, and plasmid pJQ200SK [14] were obtained from lab stocks. Strains *E*. *coli* β2155 and plasmids of pKD4 and pCVD442 were purchased from Sangon Biotech (Shanghai) Co., Ltd.

The formulation of the minimal medium used for PNP degradation was: NH_4_NO_3_ 1.0 g L^-1^, KH_2_PO_4_ 0.5 g L^-1^, K_2_HPO_4_·3H_2_O 1.96 g L^-1^, NaCl 1.0 g L^-1^, and MgSO_4_·7H_2_O 0.1 g L^-1^, pH 7.0. All strains wed used in this study could grow normal in this minimal medium.

### Construction of targeting vector and preparation of donor bacteria

The upstream and downstream homologous recombinant arms of the target gene were amplified from the genome of *Pseudomonas putida* DLL-E4, and the kanamycin resistance gene (Kn^r^) and the gentamicin resistance gene (Gm^r^) were amplified from plasmids of pKD4 and pJQ200SK, respectively. The PCR reaction systems and programs are described in S1-S5 Tables in S1 File. The upstream and downstream homologous recombinant arms corresponding to *crc*, *crcY*, and *crcZ* were ligated with Kn^r^, Kn^r^ and Gm^r^, respectively, using fusion PCR technology, to obtain targeting fragments of Δ*crc*::Kn^r^, Δ*crcY*::Kn^r^, and Δ*crcZ*::Gm^r^, respectively. The reaction systems and procedures of fusion PCR are shown in S6 and S7 Tables in S1 File. These fragments were cloned into the suicide plasmid pCVD442 to obtain the targeting vectors of pCVD442-Δ*crc*::Kn^r^, pCVD442-Δ*crcY*::Kn^r^, and pCVD442-Δ*crcZ*::Gm^r^. The vectors pCVD442-Δ*crc*::Kn^r^, pCVD442-Δ*crcY*::Kn^r^, and pCVD442-Δ*crcZ*::Gm^r^ were separately transformed into *E*.*coli* β2155 by electroporation. The products of electroporation were diluted and spread on an LB plate containing the corresponding single antibiotic, and then cultured at 37°C for the formation of single colonies. These colonies were the target donor bacteria, and were designated *E*. *coli* β2155/pCVD442-Δ*crc*::Kn^r^, *E*. *coli* β2155/pCVD442-Δ*crcY*::Kn^r^, and *E*. *coli* β2155/pCVD442-Δ*crcZ*::Gm^r^, respectively.

### Conjugation experiments and the screening of knockout strains

The recipient strain of *P*. *putida* DLL-E4 was struck from a frozen culture on a fresh LB plate and cultivated at 30°C until single colonies were formed. A single colony was selected and cultured in 3 mL liquid LB medium overnight at 30°C and 220 r min^-1^. The donor strain was cultured in 3 mL LB liquid medium containing the corresponding single antibiotic at 37°C and 220 r min^-1^ overnight. For *crc*, *crcY*, and *crcZ* knockout experiment, the donor strain is *E*. *coli* β2155/pCVD442-Δ*crc*::Kn^r^, *E*. *coli* β2155/pCVD442- Δ*crcY*::Kn^r^, and *E*. *coli* β2155/pCVD442- Δ*crcZ*::Gm^r^, respectively. After overnight cultivation, 500 μL LB culture liquid of donor strain was direct pipetted and then mixed with 500 μL that of *P*. *putida* DLL-E4 (the cells of donor strain and *P*. *putida* DLL-E4 were not washed before mixed). Once the cells were mixed, the conjugation began. A sample of 50 μL of the conjugated strain solution was spread on LB plates and cultured at 30°C until single colonies were formed. LB plate contained ampicillin and kanamycin, ampicillin and kanamycin, and ampicillin and gentamycin for the cultivation of *crc*, *crcY*, and *crcZ* conjugated strain, respectively. About 20 colonies were randomly selected and inoculated into a single tube of 100 μL LB liquid medium. After mixing well, 10 μL of the mixed solution were inoculated into 3 mL LB liquid medium, and then cultured overnight at 30°C and 220 r min^-1^. The next day, 50 μL of the culture medium was spread on an LB plate (excluding NaCl), containing 10% sucrose (10% sucrose was used to eliminate the conjugative bacteria containing plasmid pCVD442), and the corresponding single antibiotic, and then cultured at 30°C until formation of single colonies. Several colonies were randomly selected and separately cultured in 3 mL LB liquid medium containing the corresponding single antibiotic overnight at 30°C and then screened by PCR using primers designed to target internal sequences of a target gene. After the target strains were obtained, further PCR identification was performed using external primers, and the PCR products were confirmed by sequencing. Finally, the target knockout strains of *P*. *putida* DLL-Δ*crc*, *P*. *putida* DLL-Δ*crcY*, and *P*. *putida* DLL-Δ*crcZ* were obtained. The reaction procedures and systems for PCR identification are shown in S8-S11 Tables in S1 File.

### Growth curves of the original strain and the knockout strains

Single colonies of *P*. *putida* DLL-E4, DLL-Δ*crc*, DLL-Δ*crcY*, and DLL-Δ*crcZ* were inoculated into 20 mL LB liquid medium containing corresponding antibiotics, and then cultured overnight at 30°C and 180 r min^-1^. The strains were harvested the next day and diluted at 5% into 50 mL fresh LB liquid medium without any antibiotics, and then cultured at 30°C and 180 r min^-1^. Each treatment was repeated three times. The growth of each strain was monitored by OD_600_ value every 3 hours.

### PNP degradation of the original and knockout strains

#### PNP degradation using PNP as the sole carbon source

Single colonies of *P*. *putida* DLL-E4, DLL-Δ*crc*, DLL-Δ*crcY* and DLL-Δ*crcZ* were inoculated into fresh LB liquid medium containing corresponding antibiotics, and then cultured overnight at 180 r min^-1^ and 30°C. Then the cultures were inoculated (inoculum amount of 5%) into minimal medium containing 0.5 mM PNP. Each treatment was performed three times. The culture condition was set at 30°C and 180 r min^-1^. Samples were taken at regular intervals of three hours to monitor the growth and PNP degradation for each culture. The OD_600_ was monitored to evaluate growth, and OD_410_ was used to evaluate the residual concentration of PNP.

#### PNP degradation when both PNP and glucose were used as carbon source

PNP degradation with multiple carbon sources was performed according to the protocol above, but using 0.5 mM PNP and 0.25% (w:v) glucose as the carbon source in the minimal medium.

### Accession numbers

*P*. *putida* DLL-E4 has been deposited in the China Center for Type Culture Collection under collection number CCTCC AB 2015264.

## Results

### Construction of the targeting fragments

**Fig 1:**
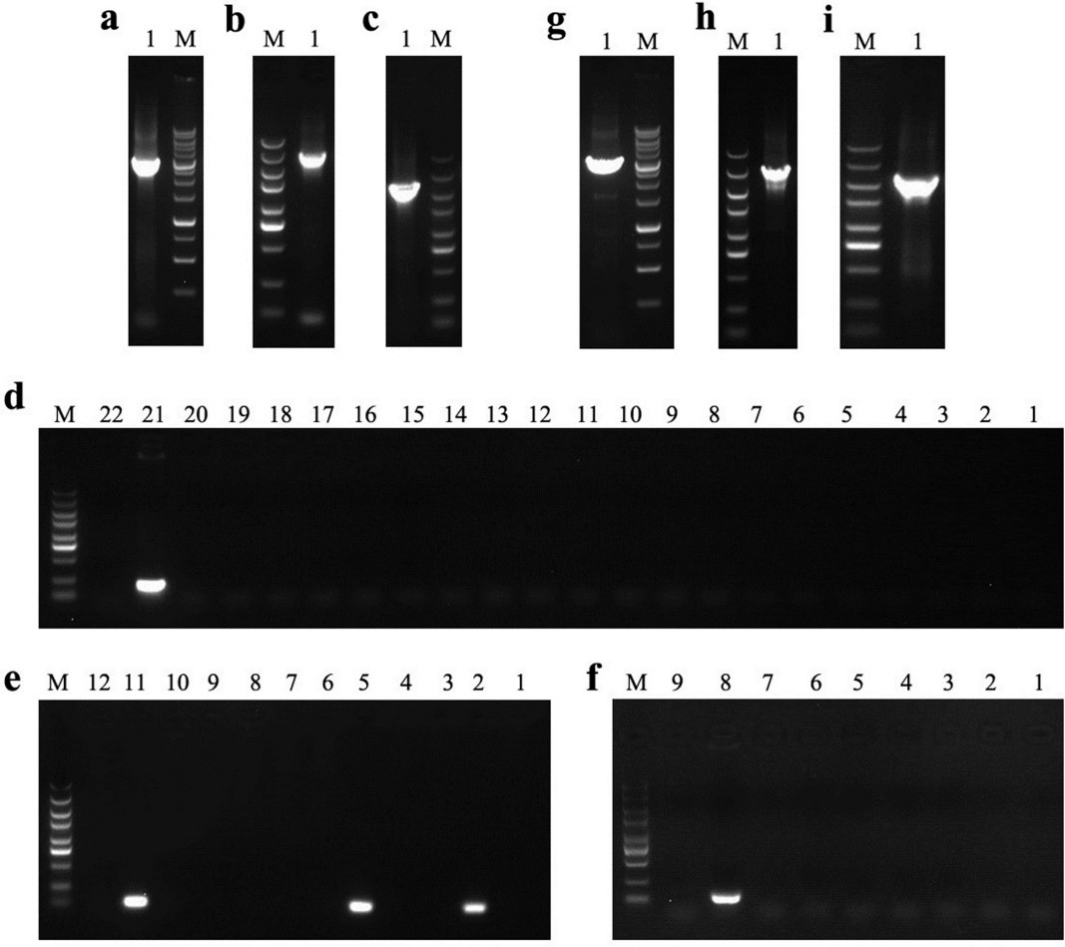
Agarose gel electrophoresis results of PCR products. Lane M: DNA molecular weight standard. (a) The fusion PCR result from the upstream and downstream homologous recombinant arms of *crc* and a kanamycin-resistant gene. Lane 1: Targeting sequences with a length of 3232 bp, containing upstream homologous recombination arm (918bp)–coding sequence of kanamycin resistant gene- downstream homologous recombination arm (847bp). (b) The fusion PCR from the upstream and downstream homologous recombinant arms of *crcY* and kanamycin-resistant gene. Lane 1: Targeting sequence with a length of 3167 bp, containing upstream homologous recombination arm (809bp) -coding sequence of kanamycin resistant gene-downstream homologous recombination arm (891bp). (c) The fusion PCR from upstream and downstream homologous recombination arms of *crcZ* and gentamicin-resistance gene. Lane 1: Targeting sequence with a length of 2611 bp, containing upstream homologous recombination arm (776bp) -coding sequence of gentamicin resistant gene-downstream homologous recombination arm (946bp). d-e: PCR products with internal identified primers in CCR gene knockout strains. (d) PCR of *crc* knockout strain. Lane 1–20: The amplification results of colonies No.1-20. If *crc* was knockout, no product could be detected; Lane 21: The amplification result of the original strain, and the length of product was 223 bp; Lane 22: The amplification result of negative control, and no product was amplified. (e) PCR of *crcY* knockout strain. Lane 1–10: The amplification products of colonies No. 1–10. If *crcY* was knockout, no product could be detected; Lane 11: The amplification results of the original strain, and the length of the product was 146 bp; Lane 12: The amplification result of negative control, and no product was amplified. (f) The PCR identification result of *crcZ* knockout strain. Lane 1–7: The amplification results of colonies No. 1–7. If *crcZ* was knockout, no product could be detected; Lane 8: The amplification results of the original strain, and the length of the product was 149 bp; Lane 9: The amplification results of negative control, and no product was amplified. g-h: PCR products using external identified primers in CCR gene knockout strains. (g) PCR identification result of *crc* knockout strain. Lane 1: The amplification result of No.1 colony. (h) The PCR product of the *crcY* knockout strain. Lane 1: The amplification result of No.1 colony. (i) The PCR identification result of *crcZ* knockout strain. Lane 1: The amplification result of No.1 colony.

To construct vectors for gene knockout, the upstream and downstream homologous recombinant arms of the target genes and related antibiotic resistance genes were successfully amplified by PCR and then joined using fusion PCR. Agarose gel electrophoresis of PCR reactions showed that the upstream and downstream homologous recombination arms of *crc*, *crcY*, and *crcZ* were successfully connected to the kanamycin resistance gene (Kn^r^) (Fig 1a), Kn^r^ (Fig 1b), and gentamicin resistant gene (Gm^r^), (Fig 1c), respectively. This confirms the correct sizes of targeting fragments (upstream homologous recombination arm—antibiotic resistance gene coding sequence—downstream homologous recombination arm) Δ*crc*::Kn^r^, Δ*crcY*::Kn^r^, and Δ*crcZ*::Gm^r^, with sequence lengths of 3232, 3167, and 2611 bp, respectively (Fig 1a–1c).

### Screening of gene knock-out strains

After confirming the targeting fragments, we cloned them separately into a suicide plasmid, pCVD442. The resulting targeting plasmids were then introduced into *E*.*coli* β2155 to obtain donor bacteria. Next, gene knock-out strains were screened using conjugation, PCR, and sequencing.

We did PCR using internal primers to preliminarily screen potential candidates for *crc* deletion. Twenty selected colonies (No. 1–20) showed no product (Fig 1d), consistent with the loss of the *crc* gene. This result was next verified using PCR with external primers. The No. 1 colony was subjected to PCR and gave a specific amplification product with the expected length (Fig 1g, theoretical amplification length of 3371 bp if *crc* was successfully replaced). Sequencing of the PCR product confirmed that the *crc* gene was successfully replaced by Kn^r^. This *crc*-knockout strain was designated *P*. *putida* DLL-Δ*crc*.

Similar to the selection for *crc*-knockout strain, PCR was performed using internal primers to preliminarily screen potential candidates for *crcY* deletion. Except for No. 2 and No. 5, the other eight colonies did not amplify a product (Fig 1e), consistent with loss of *crcY*. The No.1 colony was next subjected to PCR with external primers and a specific amplification product of expected length was obtained (Fig 1h, theoretical amplification length of 3333 bp if *crcY* was replaced). Sequencing of the PCR product showed that *crcY* was indeed successfully replaced by Kn^r^. This *crcY*-knockout strain was designated *P*. *putida* DLL-Δ*crcY*.

Seven colonies were selected as candidates for *crcZ* deletion and PCR was performed with internal primers. None of the colonies resulted in a PCR product (Fig 1f), consistent with replacement of *crcZ*. PCR with external primers was performed with the No. 1 colony, and a specific amplification product of expected length was obtained (Fig 1i, theoretical amplification length of 2783 bp if *crcZ* successfully replaced). Sequencing of the PCR product showed that *crcZ* was successfully replaced by Gm^r^. This *crcZ*-knockout strain was designated *P*. *putida* DLL-Δ*crcZ*.

### The effect of CCR knockout on growth

**Fig 2:**
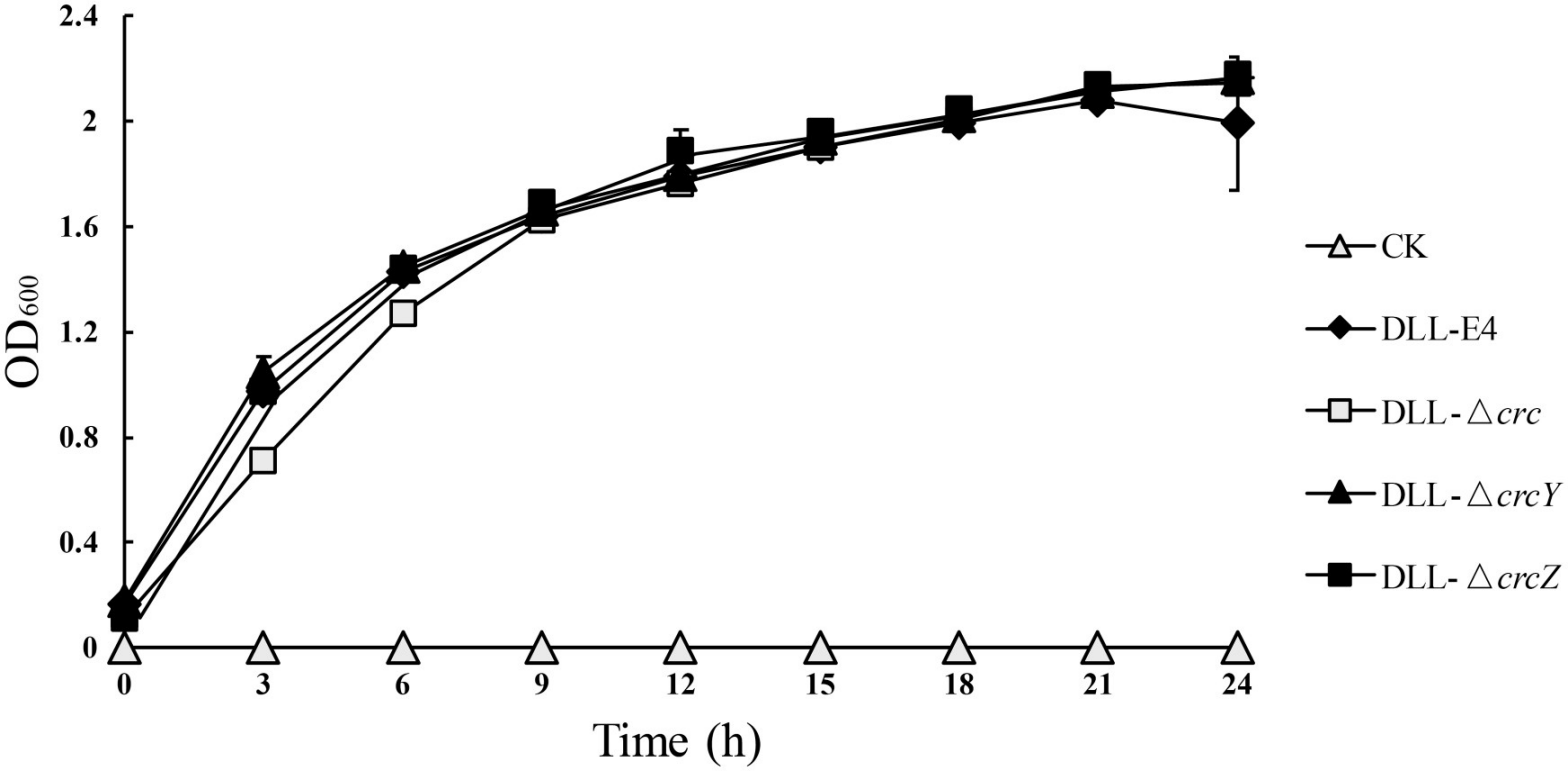
The growth of *P*. *putida* DLL-E4, DLL-Δ*crc*, DLL-Δ*crcY*, and DLL-Δ*crcZ* on LB medium. CK: control group, no inoculate any cells, to test whether the culture medium and operation are completely sterile. The values are the means of the results from three biological replicates with standard deviations. Statistical analysis indicates no differences in the growth rate among the four strains, P > 0.05.

To determine the effect of CCR-knockout on the growth of strains under complex carbon source, the growth of the four strains in LB medium was measured, and no significant difference was observed. All of them entered into the logarithmic phase within 3 h and stationary phase within 20 h, and the OD_600_ value could be up to 1.6 within 9 h (Fig 2). LB medium is a rich medium used for bacteria cultivation, and the CCR system is not expected to be required for growth in this media.

### The effect of CCR knockout on PNP degradation

**Fig 3:**
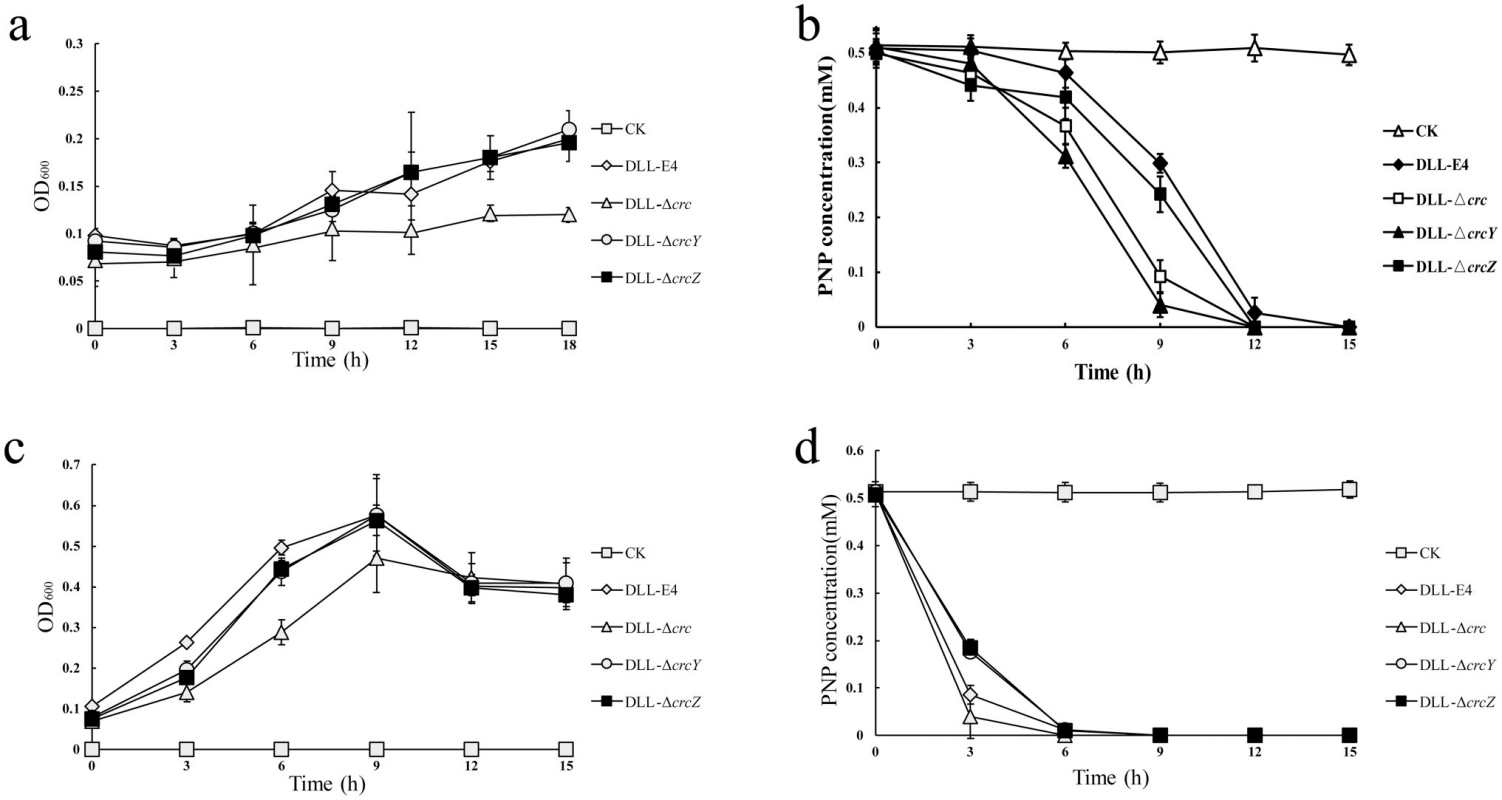
The degradation of PNP by *P*. *putida* DLL-E4, DLL-Δ*crc*, DLL-Δ*crc*Y, and DLL-Δ*crcZ*. (a) The growth of *P*. *putida* DLL-E4, DLL-Δ*crc*, DLL-Δ*crcY*, and DLL-Δ*crcZ* using PNP as the sole carbon source. (b) PNP degradation by *P*. *putida* DLL-E4, DLL-Δ*crc*, DLL-Δ*crcY*, and DLL-Δ*crcZ* using PNP used as the sole carbon source. (c) The growth of *P*. *putida* DLL-E4, DLL-Δ*crc*, DLL-Δ*crcY*, *and* DLL-Δ*crcZ* using PNP and glucose as carbon sources. (d) the degradation of PNP by *P*. *putida* DLL-E4, DLL-Δ*crc*, DLL-Δ*crcY*, and DLL-Δ*crcZ* using PNP and glucose as carbon sources. CK: control group, no inoculate any cells, to test whether the culture medium and operation are completely sterile. The values are the means of the results from three biological replicates with standard deviations.

To detect the effect of CCR knockout on the utilization of carbon sources (such as PNP), a minimal defined medium and additional carbon source (glucose) was used to cultivate the four strains. There was no significant difference in the growth among *P*. *putida* DLL-E4, DLL-Δ*crcY*, *and* DLL-Δ*crcZ* strains when PNP was used as the sole carbon source, but growth of DLL-Δ*crc* was worse than that of the other three strains (Fig 3a). After being cultivated for about 18 h, the OD_600_ value of DLL-Δ*crc* was only half of that of the other three strains. The knock-out of *crc*, *crcY*, and *crcZ* seemed accelerate the degradation rate of PNP in the former 9 h, but all of strains completely degraded PNP in around 12 h (Fig 3b). *P*. *putida* DLL-E4, *P*. *putida* DLL-Δ*crc*, *P*. *putida* DLL-Δ*crcY* and DLL-Δ*crcZ* could degrade 40.43%, 81.53%, 92.06%, and 51.63% of PNP within 9 h, respectively. When both PNP and glucose were used as carbon sources, the addition of glucose increased growth of the four strains compared to growth when PNP used as the sole carbon source. However, *P*. *putida* DLL-Δ*crc* still grew worse than *P*. *putida* DLL-E4, DLL-Δ*crcY*, and DLL-Δ*crcZ*, which showed similar growth rates (Fig 3c). Furthermore, the growth of all four strains decreased after 9h. PNP was degraded completely in advance (Fig 3d), so we speculated that the decrease in OD_600_ value after 9 h may be due to the lack of carbon source. In the presence of glucose, the rate of PNP degradation was increased for all four strains, and the degradation time of 0.5 mM PNP in all four strains was shortened from 12–15 hours to 6 hours. Although the presence of glucose decreased the difference in degradation of PNP by *P*. *putida* DLL-Δ*crc* and the wild type strain, the *crc* knockout strain still degraded PNP faster that the wild type strain. The degradation rates of PNP were in the order from fast to slow of *P*. *putida* DLL-Δ*crc*, *P*. *putida* DLL-E4, and then *P*. *putida* DLL-Δ*crcY* and DLL-Δ*crcZ* (Fig 3d). *P*. *putida* DLL-Δ*crc*, *P*. *putida* DLL-E4, *P*. *putida* DLL-Δ*crcY* and DLL-Δ*crcZ* could degrade 92.1%, 83.03%, 65.62%, and 63.75% of PNP within 3 h, respectively. When glucose was present, knockout of *crc* accelerated the degradation of PNP, but the knockout of *crcY* and *crcZ* delayed the degradation of PNP.

## Discussion

As a representative nitrophenol compound, PNP is a model pollutant to study microbial degradation. Study of the mechanism of PNP degradation regulation provides insight into microbial degradation of pollutants and microbial adaptation to the environment. The regulatory mechanism of PNP degradation by microbes has not been clearly defined. NphR, a regulatory protein belonging to the AraC/xyls family in *Rhodococcus* sp. PN1 (a PNP degrading bacterium), has a positive regulatory effect on the expression of NphA1A2 (two proteins of the PNP hydroxylase) [26]. PnpR, a LysR family regulator, modulates PNP-degradation genes *pnpA*, *pnpB*, and *pnpCDEFG* in *Pseudomonas* sp. WBC-3 at the transcriptional level [27]. Our previous work demonstrated that *pnpA* and *pnpC1C2DECX1X2*, key genes for PNP degradation in *P*. *putida* DLL-E4, are positively regulated by PnpR, a LysR family transcriptional regulator. The presence of other carbon sources (such as glucose) affects the degradation of PNP, and the expression of CCR-related genes changed accordingly [14]. Therefore, the CCR system may regulate the degradation of PNP. To test this hypothesis, *crc*, *crcY*, and *crcZ* were knocked out by conjugation experiments. The *crc*-knockout strain accelerated the degradation of PNP but slowed down the cell growth. However, the *crcY*- and *crcZ*-knockout strains accelerated PNP degradation without the presence of glucose, but they degraded PNP slower than the wildtype strain when glucose was present. This result confirms our previous hypothesis that the CCR system affects PNP degradation. However, the simple PNP degradation and only one gene knockout in this paper could not reveal the specific mechanism between CCR system and PNP degradation. We need do more experiments on the basis of the knockout strains obtained in this paper to understand the specific mechanism more systematically and deeply.

Crc inhibits the translation of genes by binding target gene mRNAs, which ultimately leads to catabolite repression [16, 17]. The knockout or inactivation of Crc eliminates catabolite repression [18, 19], and the translation of Crc target genes can proceed normally. Here, the degradation of PNP was accelerated by the knockout of *crc*, suggesting a higher expression level of PnpA in the *crc*-knockout strain than that in the original strain. Future work to understand the regulatory role of *crc* in PNP degradation should determine i) whether Crc regulates the expression of *pnpA* at the transcriptional level or at the translational level, ii) whether there is interaction between Crc and PnpA, and iii) if Crc targets *pnpA*, *pnpB*, or *pnpR*.

When the preferred carbon source was exhausted, the *crcY* and *crcZ* noncoding RNAs bound Crc and prevented Crc binding to its target genes, thus eliminating the phenomenon of catabolite repression [20, 21]. Deletion of either *crcZ* or *crcY* had no effect on catabolite repression, but the simultaneous absence of both *crcZ* and *crcY* failed to eliminate catabolite repression [21]. Here, knockout of *crcY* or *crcZ* alone had little effect on the degradation of PNP, thus we speculated that the simultaneous knockout of *crcY* and *crcZ* would make *P*. *putida* DLL-E4 unable to degrade PNP, and future work should test this. Furthermore, we need to determine how *crcY* and *crcZ* regulate PNP degradation together with *crc*, and how *crcY* and *crcZ* may affect the expression of other degradation genes in *P*. *putida* DLL-E4.

Although the knockout of *crc* would increase the rate of degradation of PNP, the *crc*-knockout strain grew more slowly than other strains on minimal medium with PNP as the sole carbon source or with PNP plus glucose as the carbon source, indicating decreased utilization of PNP metabolites by the *crc*-knockout strain. Combined with the results from our previous studies [8, 14], we suggest that this decline is due to the rapid increase of PNP denitrification products in a short period of time or due to the down-regulation of downstream degradation genes. The effect of CCR knockout on PNP degradation was complicated by the addition of glucose (Fig 3d). The presence of glucose accelerated the degradation of PNP by all strains, but the addition of glucose altered the effect of *crcY* or *crcZ* knockout on PNP degradation. The role of glucose in CCR-mediated PNP degradation remains to be further studied.

## Conclusions

To investigate the effect of the carbon catabolite repression (CCR) system on PNP degradation, the CCR Knockout strains *P*. *putida* DLL-Δ*crc*, *P*. *putida* DLL-Δ*crcY*, and *P*. *putida* DLL-Δ*crcZ* were successfully constructed by conjugation experiments. Growth experiment results showed that the CCR system is not expected to be required for growth in LB media. The further PNP degradation and growth results indicated that the knockout of *crc* accelerated PNP degradation but slowed down the cell growth, and the knockout of *crcY* or *crcZ* alone accelerated PNP degradation when PNP as the sole carbon source, but *crcY* or *crcZ* knockout alone slowed down PNP degradation when glucose was added. The results indicate that the CCR system is involved in the regulation of PNP degradation, and further work is required to determine the details of the specific regulatory mechanism.

## Supporting information

S1 File (DOCX)

S1 Raw images (PDF)
