# Supplementary material for: Preliminary study on the effect of catabolite repression gene knockout on p-nitrophenol degradation in Pseudomonas putida DLL-E4
Source: PLoS One. 2022 Dec 2;17(12):e0278503. doi: 10.1371/journal.pone.0278503 (PMC9718395; doi:10.1371/journal.pone.0278503)
Supplement: S1 File — (DOCX) [file pone.0278503.s001.docx]

**Supporting Information for**

**Preliminary Study on the Effect of Catabolite Repression Gene Knockout on *p*-nitrophenol Degradation in *Pseudomonas putida* DLL-E4**

**Shuang Li^1^，Yichao Tang^1^，Lingran Tang^1^，Xuanyu Yan^1^，Jiali Xiao^1^，Huijun Xiang^1^，Qing Wu^1^， Ruqi Yu^1^，Yushi Jin^1^，Jingyu Yu^1^，Nuo Xu^1^，Chu Wu^1^，Shengqin Wang^1^，Chuanhua Wang^1^, Qiongzhen Chen^1,2*^**

^1^ College of Life and Environmental Science, Wenzhou University, Wenzhou, People’s Republic of China

^2^ National and Local Joint Engineering Research Center for Ecological Treatment Technology of Urban Water Pollution, Wenzhou University, Wenzhou, People’s Republic of China

* Corresponding author

Email:[ashelychan@126.com](mailto:ashelychan@126.com) (QZC)

**
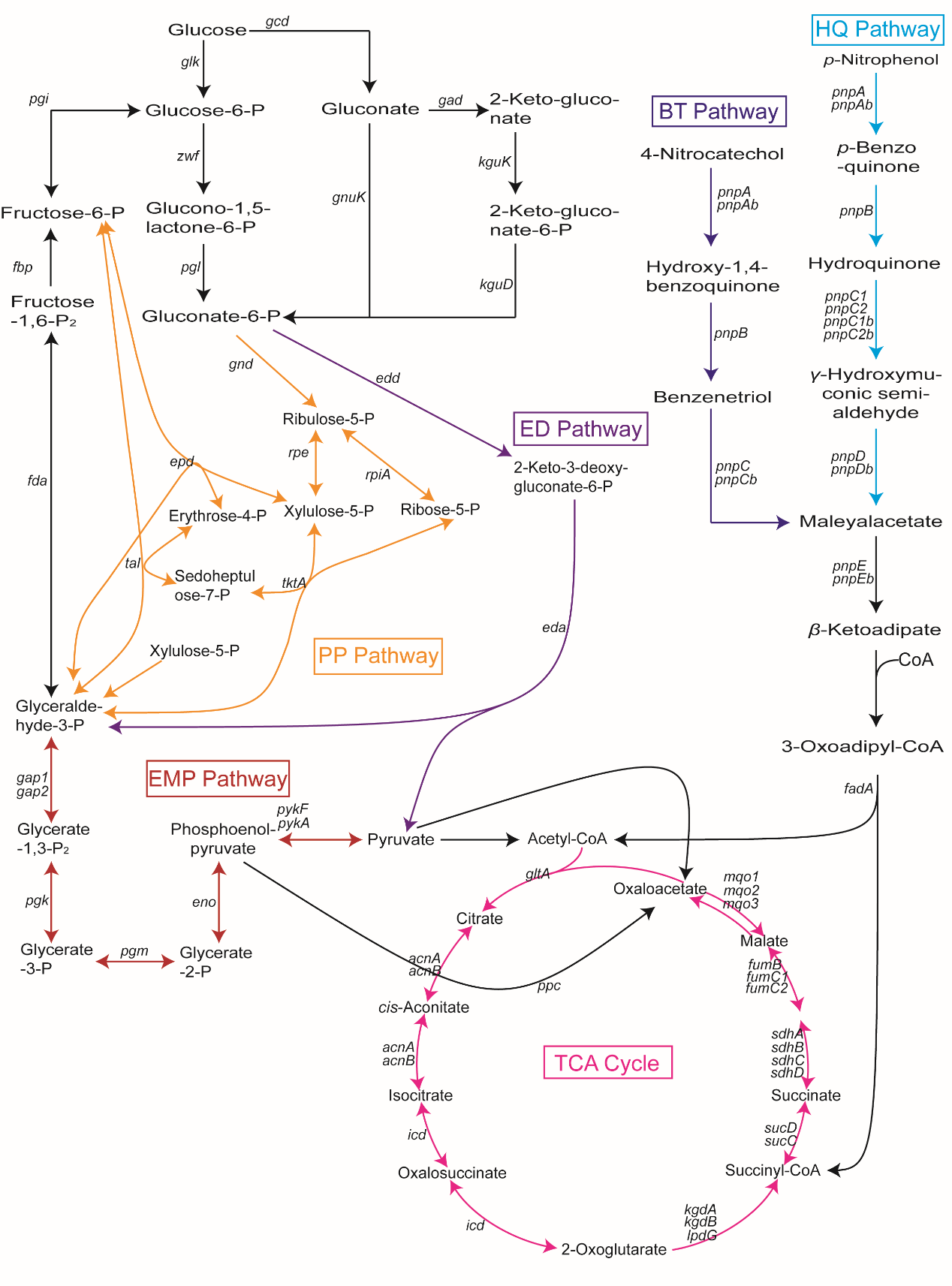
**

**Fig. S1** The relationship of glucose metabolism and PNP degradation in *Pseudomonas putida* DLL-E4.

**Table S1. The PCR reaction system used in the amplification of the upstream and downstream homologous recombination arms of *crc*, *crc*Y and *crc*Z from the genome of *Pseudomonas putida* DLL-E4.**

| For *crc* amplification | For *crcY* amplification | For *crcZ* amplification | Volume (μL) |
| --- | --- | --- | --- |
| *Pseudomonas putida* DLL-E4 culture solution | *Pseudomonas putida* DLL-E4 culture solution | *Pseudomonas putida* DLL-E4 culture solution | 0.2 |
| *crc*-5F/3F (50 pmol μL^-1^) (forward primer) | *crcY*-5F/3F2 (50 pmol μL^-1^) (forward primer) | *crcZ*-5F/3F2 (50 pmol μL^-1^) (forward primer) | 0.2 |
| *crc*-5R/3R (50 pmol μL^-1^) (reverse primer) | *crcY*-5R/3R2 (50 pmol μL^-1^) (reverse primer) | *crcZ*-5R/3R2 (50 pmol μL^-1^) (reverse primer) | 0.2 |
| dH_2_O | dH_2_O | dH_2_O | 9.4 |
| PrimeSTAR Max Premix (2×), TaKaRa | PrimeSTAR Max Premix (2×), TaKaRa | PrimeSTAR Max Premix (2×), TaKaRa | 10.0 |

**Table S2. The PCR reaction procedure used in the amplification of the upstream and downstream homologous recombination arms of *crc*, *crc*Y and *crc*Z from the genome of *Pseudomonas putida* DLL-E4.**

| No. | Temperature | Time |
| --- | --- | --- |
| 1 | 95 ℃ | 5 min |
| 2 | 95 ℃ | 30 s |
| 3 | 62 ℃ | 30 s |
| 4 | 72 ℃ | 60 s |
| 5 | from No.2 to No.4 | 10 cycles |
| 6 | 72 ℃ | 7 min |

**Table S3. The PCR reaction system used in the amplification of the coding sequence of the kanamycin-resistance gene from pKD4 plasmid.**

| For *crc* knockout | For *crcY* knockout | Volume (μL) |
| --- | --- | --- |
| pKD4（10 ng μL^-1^） | pKD4（10 ng μL^-1^） | 0.2 |
| *crc*-KnF (50 pmol μL^-1^) (forward primer) | *crcY*-KnF (50 pmol μL^-1^) (forward primer) | 0.2 |
| *crc*-KnR (50 pmol μL^-1^) (reverse primer) | *crcY*-KnR2 (50 pmol μL^-1^) (reverse primer) | 0.2 |
| dH_2_O | dH_2_O | 9.4 |
| PrimeSTAR Max Premix (2×) | PrimeSTAR Max Premix (2×) | 10.0 |

**Table S4. The PCR reaction system used in the amplification of the coding sequence of the gentamicin-resistance gene from pJQ200SK plasmid.**

| Reagent | Volume (μL) |
| --- | --- |
| pJQ200SK（10 ng μL^-1^） | 0.2 |
| *crcZ*-GmF (50 pmol μL^-1^) (forward primer) | 0.2 |
| *crcZ*-GmR2 (50 pmol μL^-1^) (reverse primer) | 0.2 |
| dH_2_O | 9.4 |
| PrimeSTAR Max Premix (2×) | 10.0 |
| Total | 20.0 |

**Table S5. The PCR reaction procedure used in the amplification of the coding sequence of the kanamycin-resistance gene and the gentamicin-resistance gene from plasmids pKD4 and pJQ200SK, respectively.**

| No. | Temperature | Time |
| --- | --- | --- |
| 1 | 95 ℃ | 5 min |
| 2 | 95 ℃ | 30 s |
| 3 | 58 ℃ | 30 s |
| 4 | 72 ℃ | 60 s |
| 5 | from No.2 to No.4 | 10 cycles |
| 6 | 72 ℃ | 7 min |

**Table S6. The fusion PCR reaction system for obtaining gene-knockout targeting fragments.**

| For *crc* | For *crcY* | For *crcZ* | Volume (μL) |
| --- | --- | --- | --- |
| *crc*-5F/5R PCR product | *crcY*-5F/5R PCR product | *crcZ*-5F/5R PCR product | 3.2 |
| *crc*-3F/3R PCR product | *crcY*-3F2/3R2 PCR product | *crcZ*-3F2/3R2 PCR product | 3.2 |
| *crc*-KnF/R PCR product | *crcY*-KnF/R2 PCR product | *crcZ*-GmF/R2 PCR product | 3.2 |
| *crc*-5F (50 pmol μL^-1^) | *crcY*-5F (50 pmol μL^-1^) | *crcZ*-5F (50 pmol μL^-1^) | 0.2 |
| *crc*-3R (50 pmol μL^-1^) | *crcY*-3R (50 pmol μL^-1^) | *crcZ*-3R2 (50 pmol μL^-1^) | 0.2 |
| PrimeSTAR Max Premix (2×) | PrimeSTAR Max Premix (2×) | PrimeSTAR Max Premix (2×) | 10.0 |

**Table S7. The fusion PCR reaction procedure for obtaining gene-knockout targeting fragments.**

| No. | Temperature | Time |
| --- | --- | --- |
| 1 | 95 ℃ | 5 min |
| 2 | 95 ℃ | 30 s |
| 3 | 58 ℃ | 30 s |
| 4 | 72 ℃ | 2.5 min |
| 5 | from No.2 to No.4 | 25 cycles |
| 6 | 72 ℃ | 7 min |

**Table S8. The internal identified PCR reaction system for screening gene-knockout strains.**

| For *crc* | For *crcY* | For *crcZ* | Volume (μL) |
| --- | --- | --- | --- |
| selected strain culture solution | selected strain culture solution | selected strain culture solution | 0.5 |
| 10× Taq buffer | 10× Taq buffer | 10× Taq buffer | 5.0 |
| dNTP (2.5 mM) | dNTP (2.5 mM) | dNTP (2.5 mM) | 4.0 |
| *crc*-inF (50 pmol μL^-1^) | *crcY*-inF (50 pmol μL^-1^) | *crcZ*-inF (50 pmol μL^-1^) | 0.5 |
| *crc*-inR (50 pmol μL^-1^) | *crcY*-inR (50 pmol μL^-1^) | *crcZ*-inR (50 pmol μL^-1^) | 0.5 |
| Taq DNA polymerase (5 U μL^-1^) | Taq DNA polymerase (5 U μL^-1^) | Taq DNA polymerase (5 U μL^-1^) | 0.5 |
| dH_2_O | dH_2_O | dH_2_O | 39.0 |

**Table S9. The internal identified PCR reaction procedure for screening gene-knockout strains.**

| No. | Temperature | Time |
| --- | --- | --- |
| 1 | 95 ℃ | 5 min |
| 2 | 95 ℃ | 30 s |
| 3 | *crc*: 64 ℃; *crcY*: 62℃; *crcZ*: 58℃ | 30 s |
| 4 | 72 ℃ | 30 s |
| 5 | from No.2 to No.4 | 30 cycles |
| 6 | 72 ℃ | 7 min |

**Table S10. The external identified PCR reaction system for screening gene-knockout strains.**

| For *crc* | For *crcY* | For *crc*Z | Volume (μL) |
| --- | --- | --- | --- |
| selected strain culture solution | selected strain culture solution | selected strain culture solution | 0.5 |
| 10× TaqPlus buffer | 10× TaqPlus buffer | 10× TaqPlus buffer | 5.0 |
| dNTP (2.5 mM) | dNTP (2.5 mM) | dNTP (2.5 mM) | 4.0 |
| *crc*-outF (50 pmol μL^-1^) | *crcY*-outF (50pmol μL^-1^) | *crcZ*-outF (50 pmol μL^-1^) | 0.5 |
| *crc*-outR (50 pmol μL^-1^) | *crcY*-outR (50 pmol μL^-1^) | *crcZ*-outR (50 pmol μL^-1^) | 0.5 |
| TaqPlus DNA polymerase (5 U μL^-1^) | TaqPlus DNA polymerase (5 U μL^-1^) | TaqPlus DNA polymerase (5 U μL^-1^) | 0.5 |
| dH_2_O | dH_2_O | dH_2_O | 39.0 |

**Table S11. The external identified PCR reaction procedure for screening gene-knockout strains.**

| No. | Temperature | Time |
| --- | --- | --- |
| 1 | 95 ℃ | 5 min |
| 2 | 95 ℃ | 30 s |
| 3 | 62 ℃ | 30 s |
| 4 | 72 ℃ | 3.5 min |
| 5 | from No.2 to No.4 | 35 cycles |
| 6 | 72 ℃ | 7 min |
