# Supplementary material for: Preliminary study on the effect of catabolite repression gene knockout on p-nitrophenol degradation in Pseudomonas putida DLL-E4
Source: PLoS One. 2022 Dec 2;17(12):e0278503. doi: 10.1371/journal.pone.0278503 (PMC9718395; doi:10.1371/journal.pone.0278503)
Supplement: S1 Raw images — (PDF) [file pone.0278503.s002.pdf]

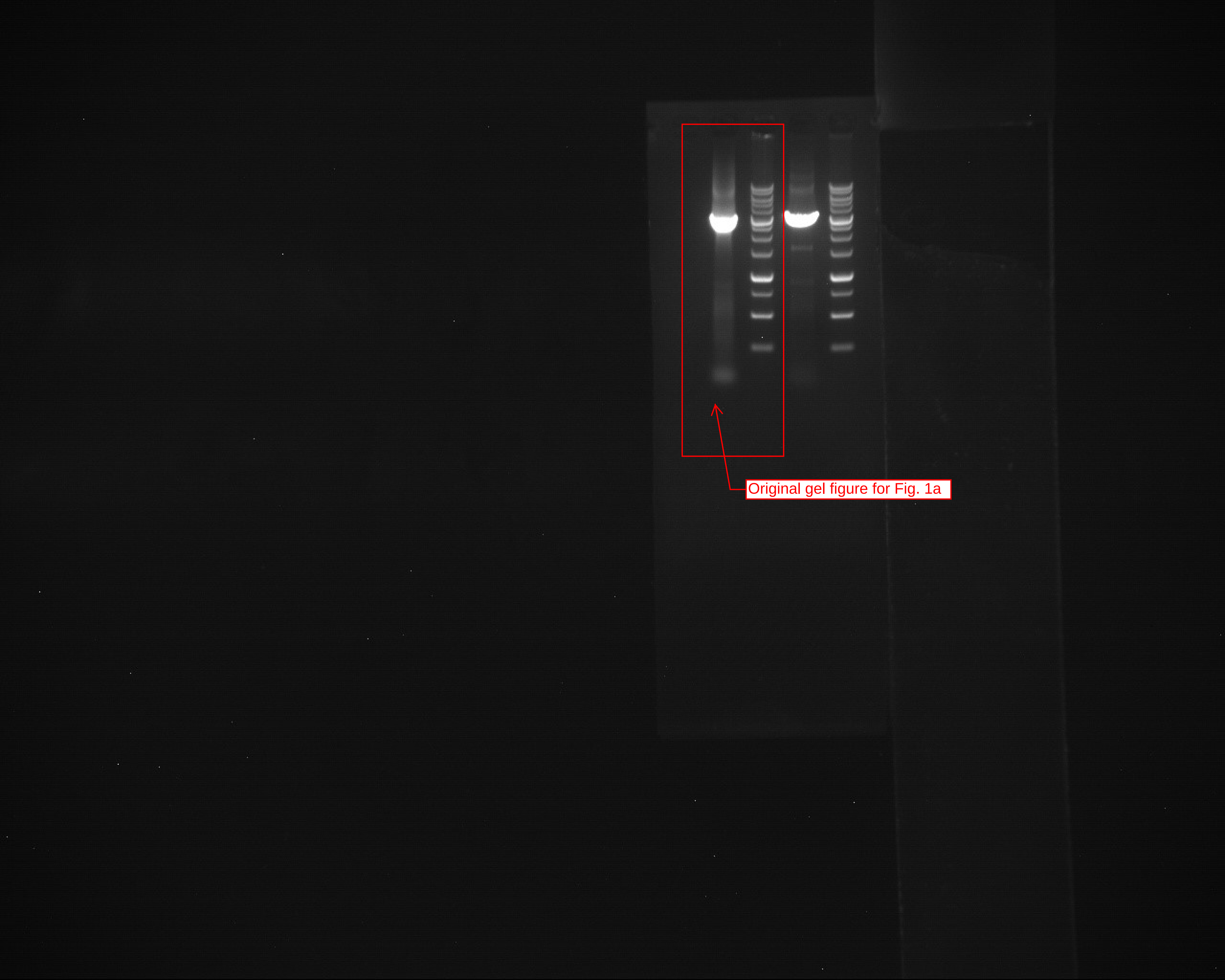
Original gel figure for Fig. 1a

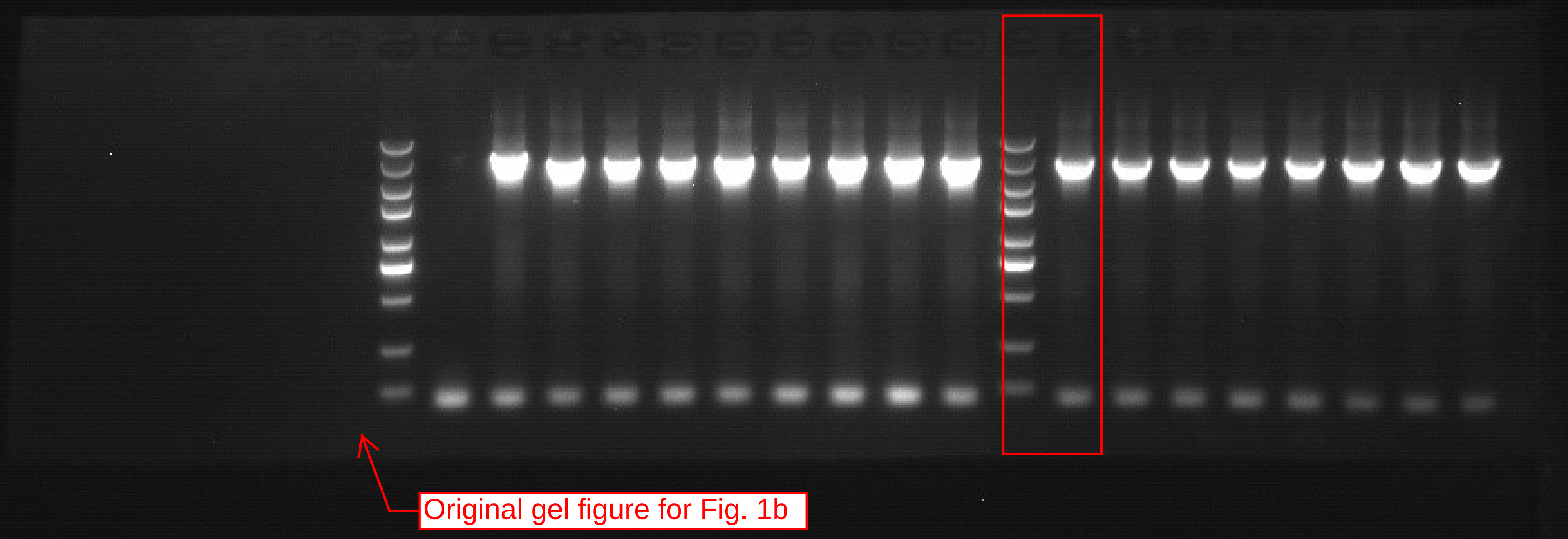
Original gel figure for Fig. 1b

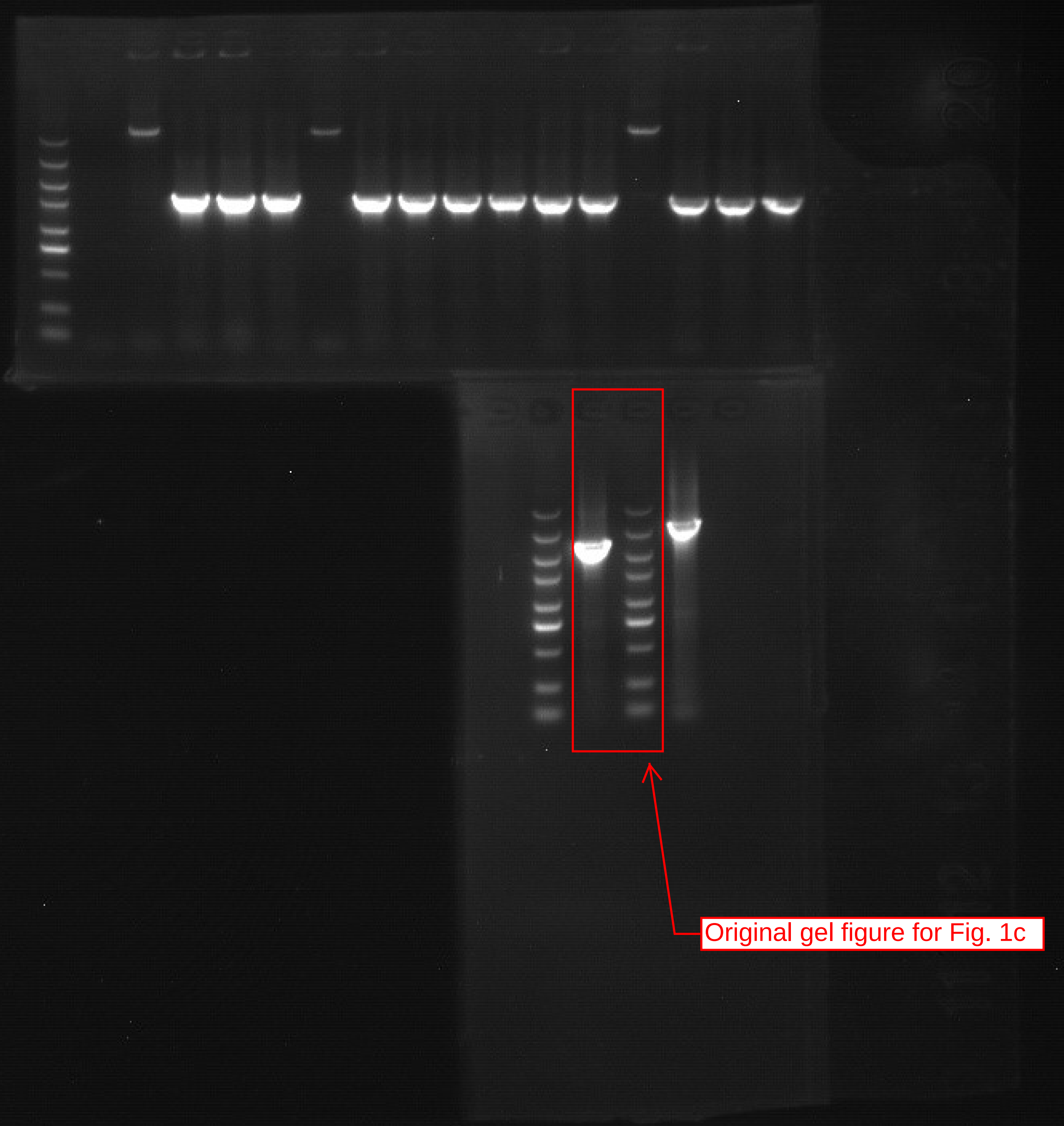
Original gel figure for Fig. 1c

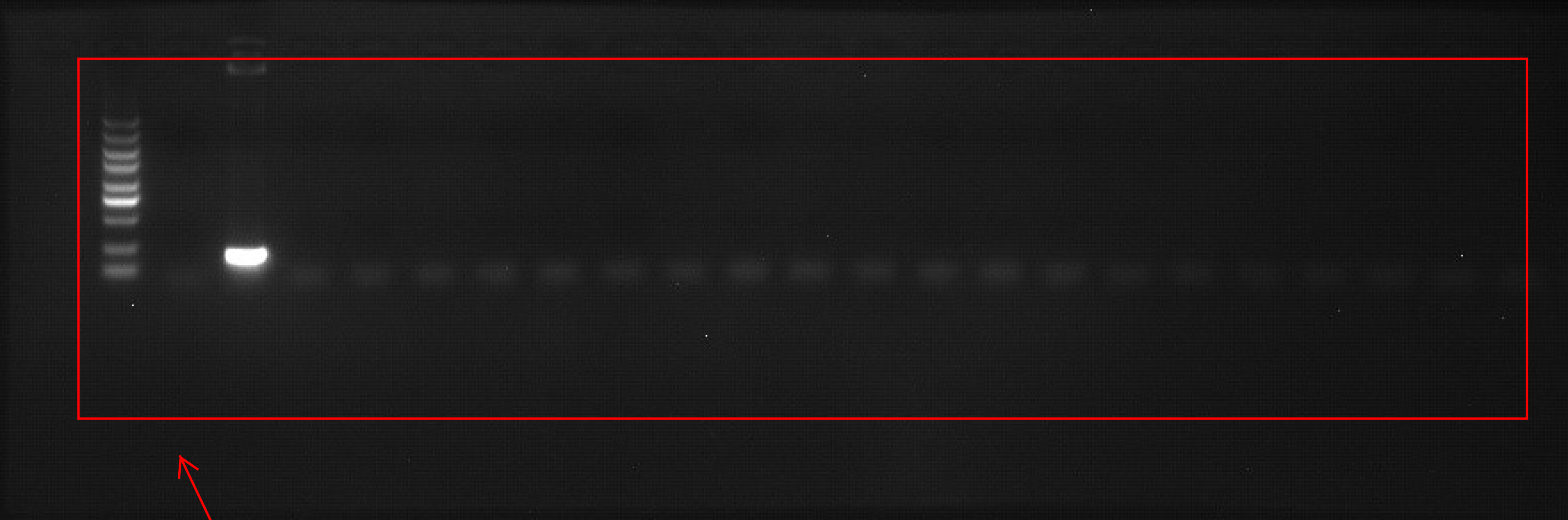

Original gel figure for Fig. 1d

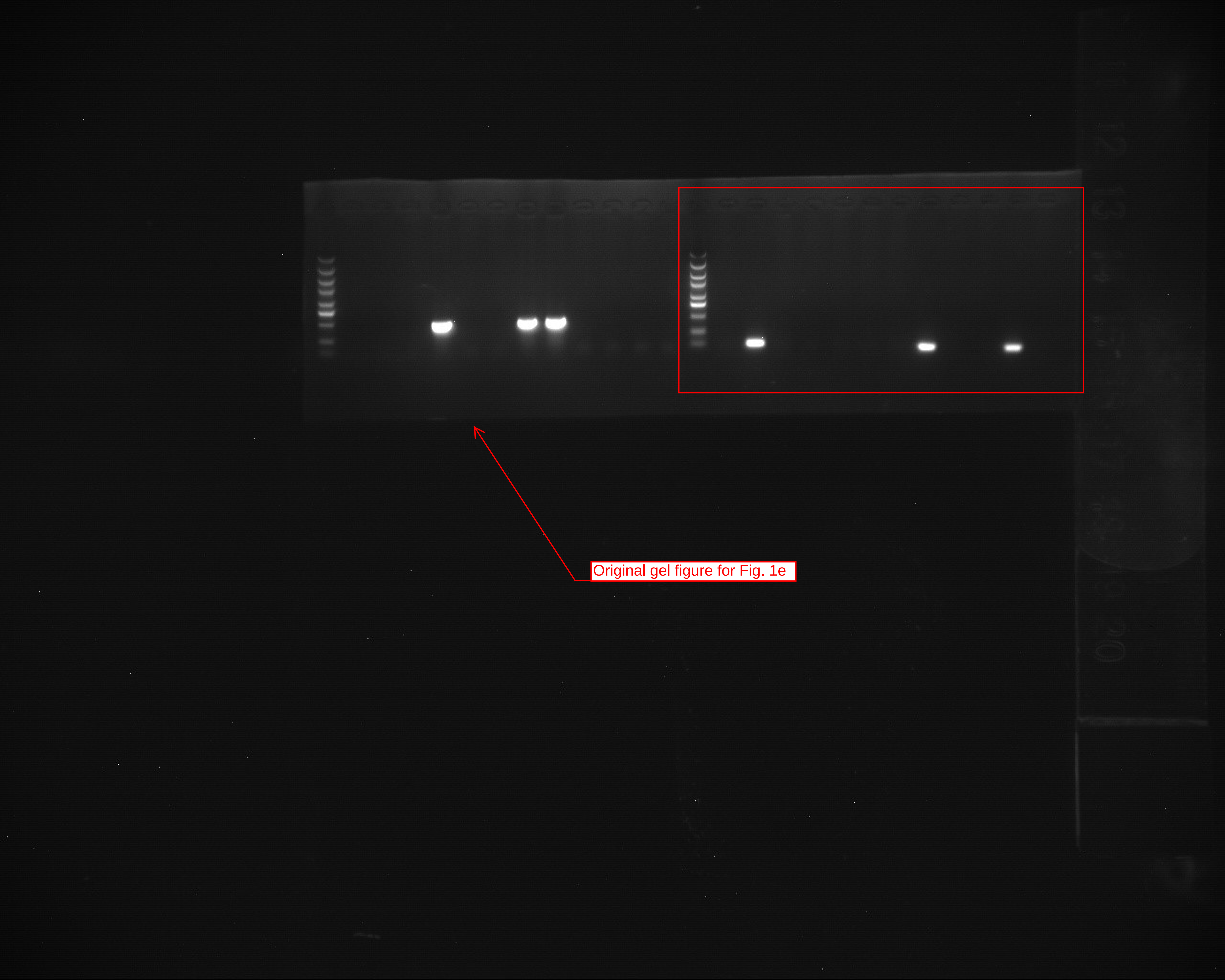
Original gel figure for Fig. 1e

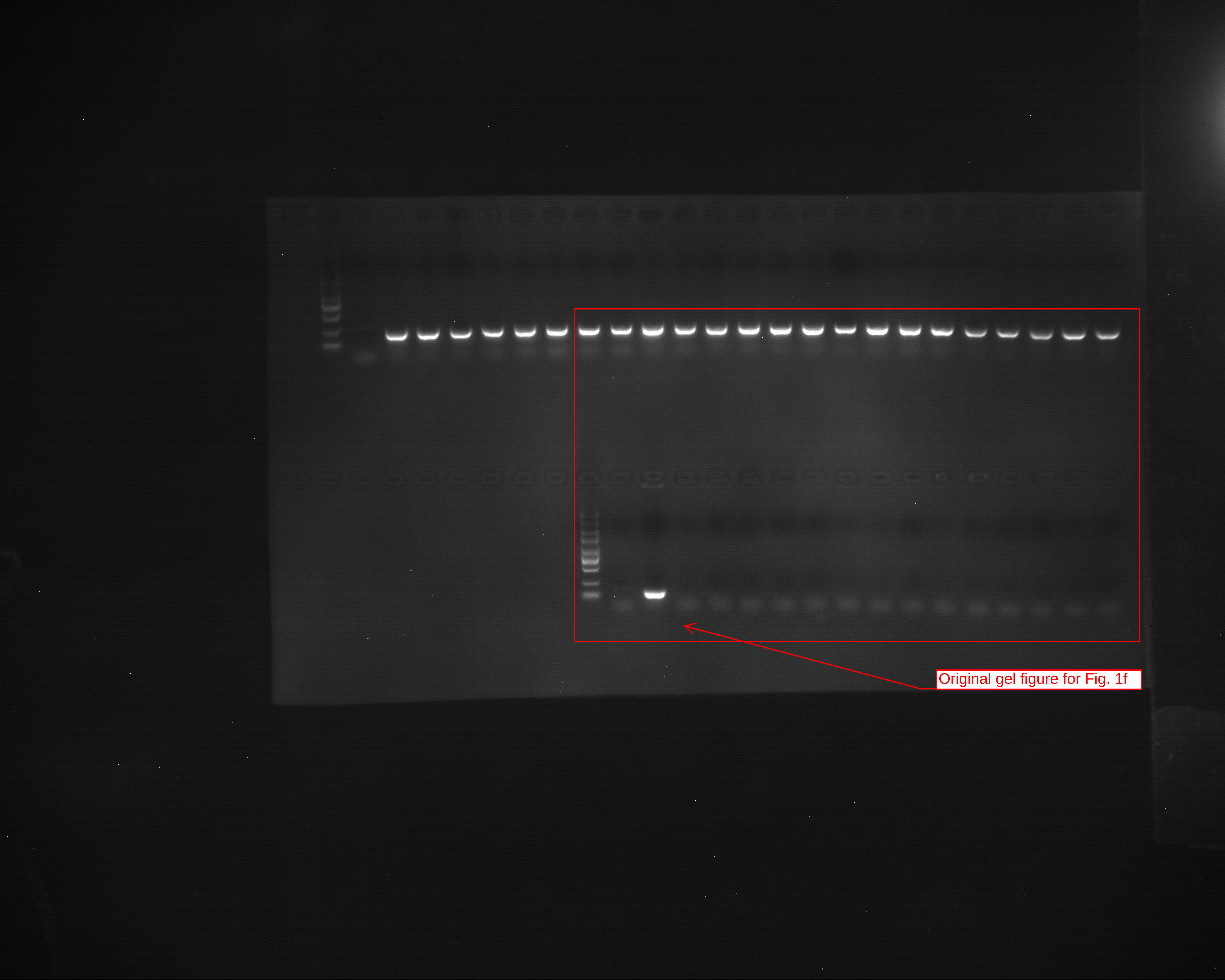
Original gel figure for Fig. 1f

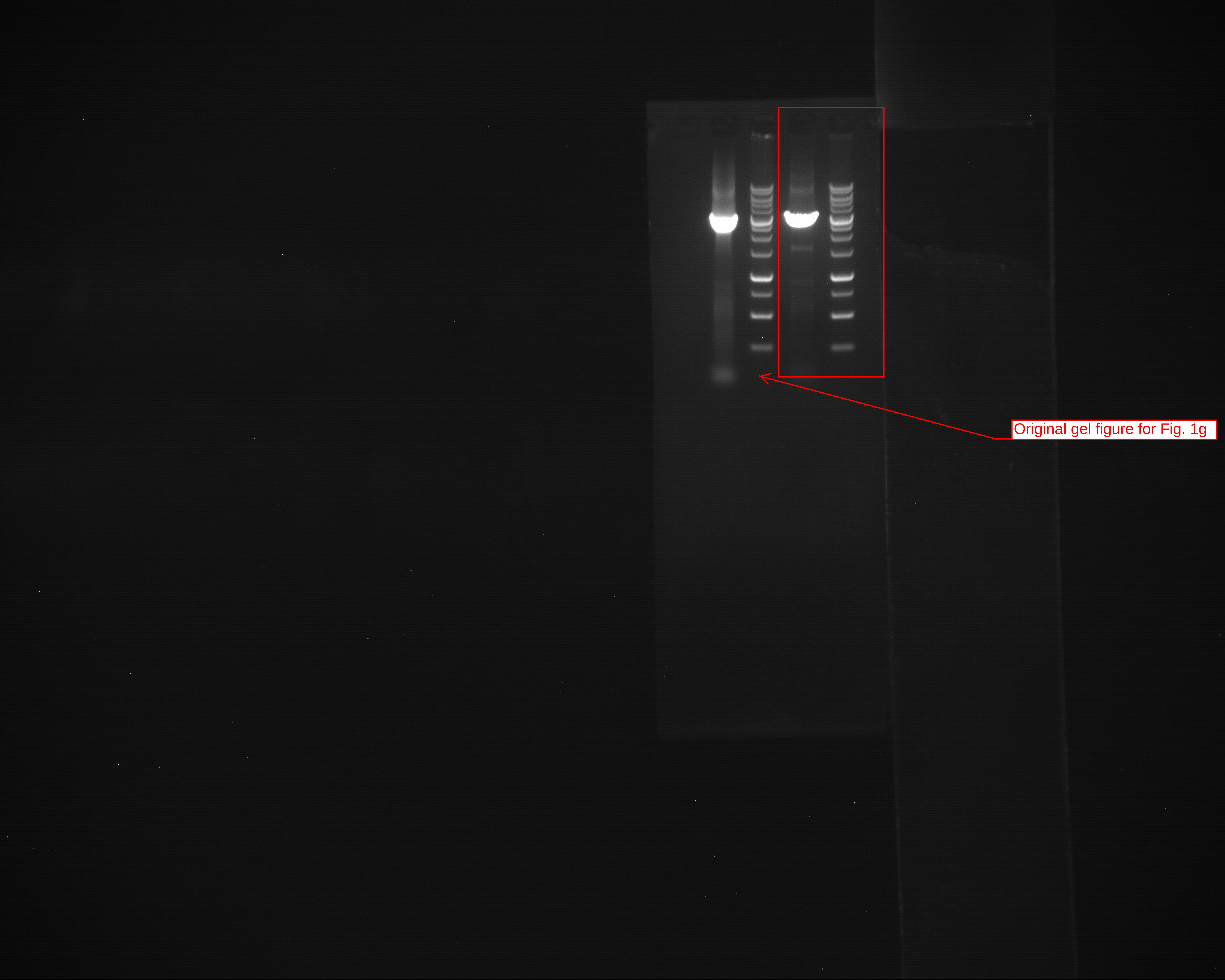
Original gel figure for Fig. 1g

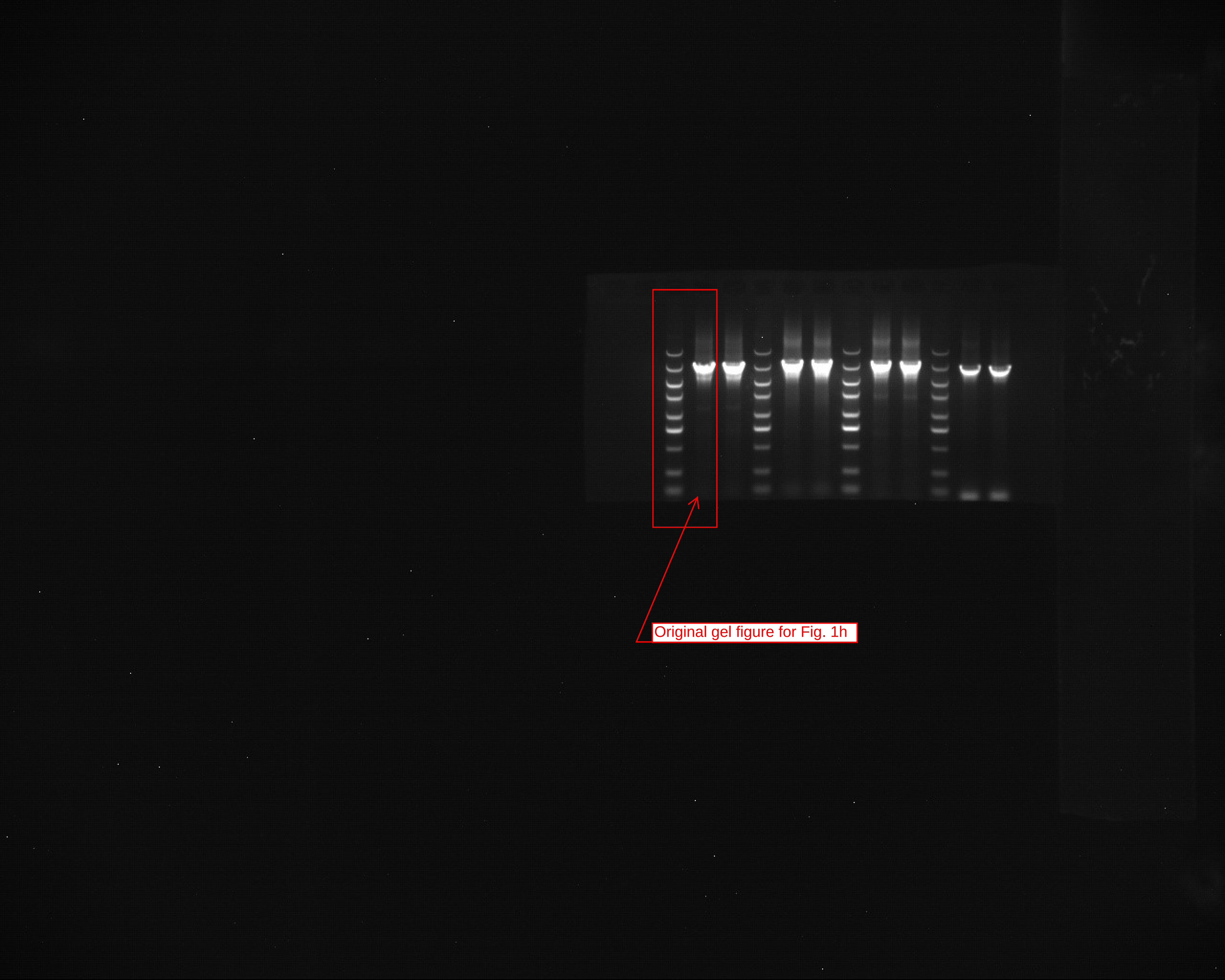
Original gel figure for Fig. 1h

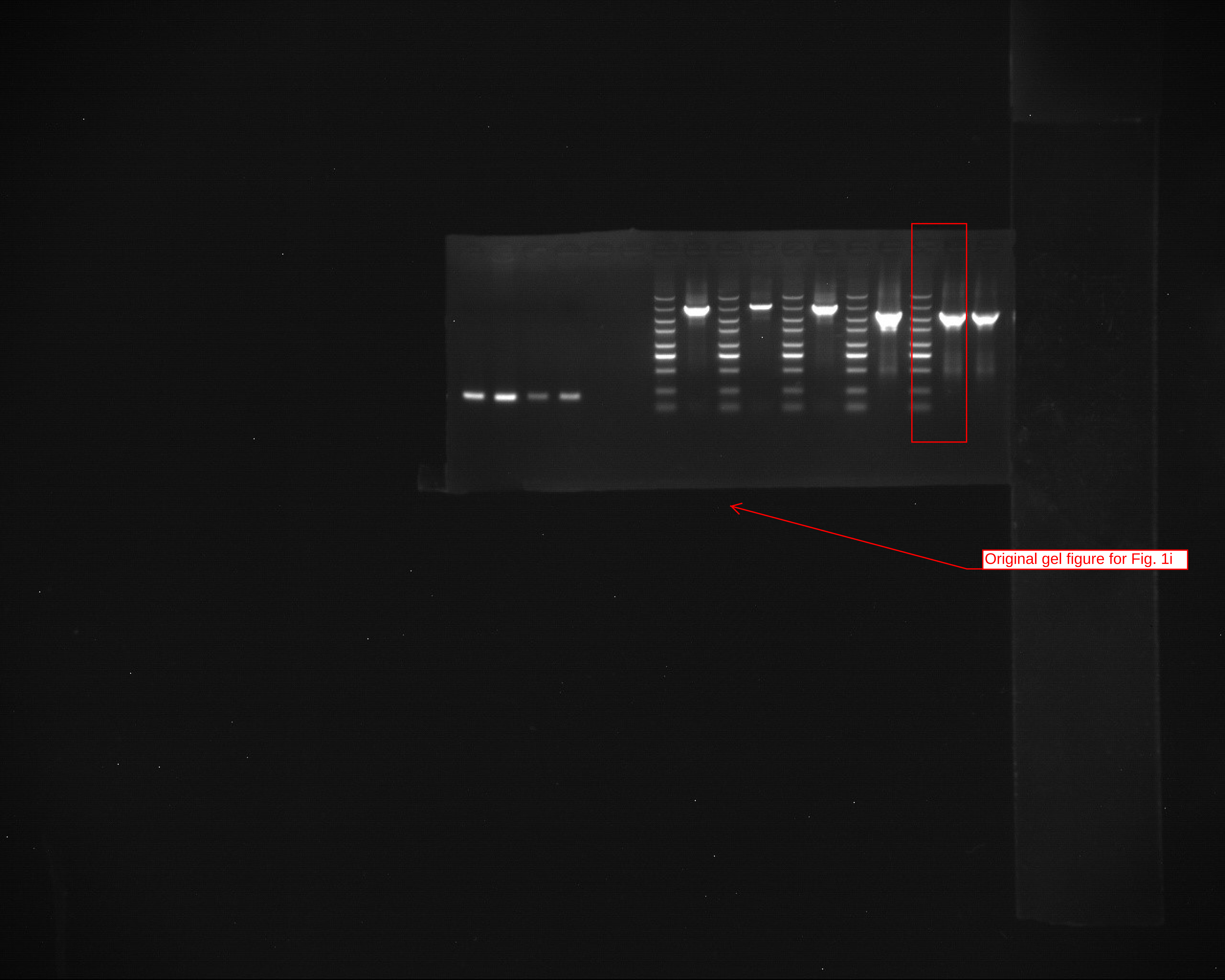
Original gel figure for Fig. 1i
